# Importance of Imaging Assessment Criteria in Predicting the Need for Post-Dilatation in Transcatheter Aortic Valve Implantation with a Self-Expanding Bioprosthesis

**DOI:** 10.3390/jcdd12080296

**Published:** 2025-08-01

**Authors:** Matthias Hammerer, Philipp Hasenbichler, Nikolaos Schörghofer, Christoph Knapitsch, Nikolaus Clodi, Uta C. Hoppe, Klaus Hergan, Elke Boxhammer, Bernhard Scharinger

**Affiliations:** 1 Department of Internal Medicine II, Division of Cardiology, Paracelsus Medical University of Salzburg, 5020 Salzburg, Austria; philipp.hasenbichler@stud.pmu.ac.at (P.H.); u.hoppe@salk.at (U.C.H.); e.boxhammer@salk.at (E.B.); 2 Department of Radiology, Paracelsus Medical University of Salzburg, 5020 Salzburg, Austria; n.schoerghofer@salk.at (N.S.); c.knapitsch@salk.at (C.K.); k.hergan@salk.at (K.H.);

**Keywords:** aortic valve stenosis, computed tomography, echocardiography, post-dilatation, TAVI

## Abstract

Background: Transcatheter aortic valve implantation (TAVI) has revolutionized the treatment of severe aortic valve stenosis (AS). Balloon post-dilatation (PD) remains an important procedural step to optimize valve function by resolving incomplete valve expansion, which may lead to paravalvular regurgitation and other potentially adverse effects. There are only limited data on the predictors, incidence, and clinical impact of PD during TAVI. Methods: This retrospective, single-center study analyzed 585 patients who underwent TAVI (2016–2022). Pre-procedural evaluations included transthoracic echocardiography and CT angiography to assess key parameters, including the aortic valve calcium score (AVCS); aortic valve calcium density (AVCd); aortic valve maximal systolic transvalvular flow velocity (AV Vmax); and aortic valve mean systolic pressure gradient (AV MPG). We identified imaging predictors of PD and evaluated associated clinical outcomes by analyzing procedural endpoints (according to VARC-3 criteria) and long-term survival. Results: PD was performed on 67 out of 585 patients, with elevated AV Vmax (OR: 1.424, 95% CI: 1.039–1.950; *p* = 0.028) and AVCd (OR: 1.618, 95% CI: 1.227–2.132; *p* = 0.001) emerging as a significant independent predictor for PD in TAVI. Kaplan–Meier survival analysis revealed no significant differences in short- and mid-term survival between patients who underwent PD and those who did not. Interestingly, patients requiring PD exhibited a lower incidence of adverse events regarding major vascular complications, permanent pacemaker implantations and stroke. Conclusions: The study highlights AV Vmax and AVCd as key predictors of PD. Importantly, PD was not associated with increased procedural adverse events and did not predict adverse events in this contemporary cohort.

## 1. Introduction

Transcatheter aortic valve implantation (TAVI) has become a pivotal therapeutic approach for patients with severe aortic stenosis (AS), offering a minimally invasive alternative to surgery, characterized by reduced recovery times and expanded applicability to older patients (e.g., >75 years), regardless of surgical risk, and patients who are ineligible for surgical intervention [1,2,3]. Despite the procedural advancements and improvements in patient outcomes, certain technical challenges remain, one of which is the need for post-dilatation (PD) after initial valve deployment [4].

PD in TAVI is an adjunctive step involving balloon expansion of the implanted prosthetic valve to correct suboptimal results from the initial valve placement. This procedure is primarily employed to address issues such as residual paravalvular regurgitation (PVR), incomplete valve expansion, or malpositioning [5]. While PD can optimize valve function by improving seating and reducing PVR, it is associated with several risks, including extended procedural duration, increased likelihood of cerebrovascular events, conduction abnormalities, valve embolization, and even annular rupture [5,6]. As a result, identifying the factors that drive the necessity for PD might have an impact on patient selection, procedural planning, and clinical outcomes. The reported frequency of PD in TAVI procedures varies widely, with rates ranging from 10% to 40% [5,6]. This variation can be attributed to differences in patient populations, valve designs, operator experience, and criteria for procedural success.

Echocardiography and multi-detector computed tomography (MDCT) are indispensable to evaluating the structural and functional characteristics of the aortic valve complex and the access vasculature before TAVI [7,8]. Key parameters, including the aortic valve calcification score (AVCS), annular dimensions, and left ventricular outflow tract (LVOT) morphology, could be crucial in predicting the likelihood of residual PVR and the requirement for PD. For instance, a high AVCS could potentially be associated with incomplete valve expansion and persistent PVR, making it a significant predictor for PD [9]. Additionally, the degree of leaflet calcification, as visualized by echocardiography and MDCT, could influence valve expansion dynamics [10].

Our retrospective study has two main objectives:

1. It aims to assess the incidence of PD in patients undergoing transfemoral TAVI with a self-expandable system and to identify imaging criteria derived from echocardiography and computed tomography that may causally contribute to, and therefore predict, the need for balloon PD;
2. We evaluate the clinical impact of PD by analyzing procedural outcomes and long-term all-cause mortality.

## 2. Materials and Methods

### 2.1. Study Cohort

This study included a cohort of 585 individuals who were eligible for TAVI at Paracelsus Medical University Hospital in Salzburg between 2016 and 2022. Patient eligibility for TAVI was determined by a multidisciplinary heart team comprising experts in cardiac surgery, interventional cardiology, and anesthesiology. The patient selection process for the study cohort was both retrospective and consecutive.

### 2.2. Ethics Declaration

The research followed ethical guidelines in accordance with the Declaration of Helsinki and Good Clinical Practice. Approval for the study was granted by the Ethics Commission of the State of Salzburg (Land Salzburg Ethikkommision, EK-Nr. 1082/2024). The informed consent requirement was waived by the ethics committee as this is a retrospective analysis using deidentified data.

### 2.3. Data Collection

Patient data were retrieved from the ORBIS electronic medical records system (Agfa Healthcare, Version 08043301.04110DACHL) and from a separate medical archive system (Krankengeschichtsarchiv System, Uniklinikum Salzburg, Softworx by Andreas Schwab^TM^, 2008). The collected information included patient charts, hospital admission and discharge summaries, as well as echocardiography and imaging reports associated with the TAVI procedures.

### 2.4. Transthoracic Echocardiography (TTE)

Routine transthoracic echocardiograms were performed 1 to 4 weeks prior to TAVI, using either the iE33 or Epiq 5 ultrasound systems (Philips Healthcare, Hamburg, Germany). These scans were conducted by clinicians with over 4 years of echocardiography experience. Severe aortic stenosis was classified in line with current European Society of Cardiology (ESC) guidelines, which define severity based on specific echocardiographic parameters, including an aortic valve maximum velocity (AV Vmax) of ≥4.0 m/s; an aortic valve maximum pressure gradient (AV MAX) of ≥60 mmHg; an aortic valve mean pressure gradient (AV MPG) of ≥40 mmHg; and a left ventricular ejection fraction (LVEF) of ≥50%. LVEF was determined using Simpson’s method.

### 2.5. Computed Tomography Angiography and Calcium Scoring

Before intervention, patients underwent routine ECG-gated non-contrast computed tomography (CT) of the heart to assess the aortic valve calcium and ECG-gated CT angiography (CTA) of the entire aorta down to the femoral arteries. These scans evaluated the aortic annulus (measuring diameter, perimeter, and area), aortic valve calcium burden, vascular anatomy, and potential access routes. The imaging was performed using either 128- or 256-slice dual-source CT scanners (Revolution, General Electric Healthcare, Chicago, IL, USA or Somatom Definition AS+, Siemens Healthcare, Erlangen, Germany), with the tube voltage (80–120 kVp) and current modulation adjusted for patient size. A bolus of 100 mL of non-ionic iodinated contrast medium, followed by 70 mL of saline, was administered at a flow rate of 3.5–5 mL/s using bolus-tracking technology.

The Agatston score for aortic valve calcium (AVCS) and annulus measurements were determined by a radiologist who specialized in cardiovascular imaging using dedicated software (Impax 6, Agfa-Gevaert, Mortsel, Belgium; IntelliSpace, Philips, Amsterdam, The Netherlands). The AVCS was calculated by identifying calcified areas on non-contrast CT and applying the Agatston method, which multiplies the plaque area by a density factor based on the highest attenuation. Aortic annulus measurements were taken during mid-systole, with imaging planes manually adjusted to align with the annulus orientation. Semiautomated tools calculated annulus perimeter, diameter, and area, which were critical in selecting the appropriate TAVI prosthesis. AVCS was indexed to the patient’s body surface area (BSA), calculated using the Dubois formula, as follows: BSA = 0.007184 × height 0.725 × weight 0.425, receiving the aortic valve calcium index (AVCi). AVCS was also indexed on the annulus area by calculating the aortic valve calcium density (AVCd).

### 2.6. TAVI Procedure and PD

All 585 patients underwent transfemoral TAVI using either second-generation (CoreValve™ Evo-lut™ R; Medtronic Inc., Minneapolis, MN, USA) or third-generation (CoreValve™ Evolut™ Pro; Medtronic Inc., Minneapolis, MN, USA) devices. The procedure followed standard protocols. Diagnosis of AS, indication for valve replacement, anatomical measurements, and vascular access were determined by pre-procedural imaging, including TTE, CT, and, when needed, transesophageal echo-cardiography (TEE). Aortic annulus measurements were used for choosing the correct valve size according to the instructions for use.

The indication for PD was determined jointly by the implanting physicians within minutes after deployment of the prosthesis, taking into account the following criteria:

1. Structural assessment of the valve prosthesis using rotational fluoroscopy: insufficient spontaneous expansion affecting the entire circumference (“underexpansion”) and/or localized infolding;
2. Functional assessment using angiography and instantaneous transvalvular pressure measurement: unacceptable paravalvular leakage and/or residual stenosis.

The following criteria were weighed against the risk of potential complications of PD and other objections to PD:

1. Valve dislocation/embolization: implantation height;
2. Annulus rupture: eccentric sub-/supra-/valvular calcifications;
3. Hemodynamic intolerance of rapid ventricular pacing: left ventricular dysfunction, arrhythmia;
4. Lack of benefit from PD: concomitant (coronary, valvular) heart disease or extracardiac disease, individual life expectancy, age.

Balloon PD was performed using a compliant balloon catheter (Osypka, OSYPKA AG, Rheinfelden, Germany). Balloon size was selected based on the size of the implanted valve and additional structural criteria (primarily the size of the anatomical annulus and valvular/subvalvular calcification). Balloon valvuloplasty was performed at the valve site under fluoroscopic guidance and rapid right ventricular pacing by temporary transvenous pacemaker or—if present—permanent pacemaker. It was carried out according to the instructions for use by short-time manual (i.e., “low pressure”) inflation (up to approximately 1 s) without a manometer. Therefore, the applied maximum pressure and the exact duration of maximum balloon inflation could not be assessed quantitatively.

### 2.7. Clinical Outcomes

The primary outcome of the study was overall survival, measured from the time of TAVI to a two-year follow-up. This outcome was used to assess the mid-term effectiveness of the procedure in extending the life expectancy of patients and the potential impact of PD on this. Secondary safety outcomes included the incidence of procedural adverse events, such as major vascular complications, defined according to the current Valve Academic Research Consortium (VARC-3) criteria [11] as: severe bleeding requiring vascular surgical intervention; large vessel perforation or dissection; significant bleeding necessitating the transfusion of four or more units of blood; and distal embolization causing irreversible end-organ damage. Additional secondary outcomes included the need for permanent pacemaker implantation and cerebrovascular events such as stroke.

### 2.8. Statistical Analysis

All statistical analyses and data visualizations were conducted using SPSS software (Version 25.0, SPSS Inc., Armonk, NY, USA). To assess the distribution of variables, the Kolmogorov–Smirnov–Lilliefors test was applied. Continuous variables following a normal distribution were reported as mean ± standard deviation (SD) and compared using an unpaired Student’s *t*-test. For non-normally distributed variables, data were expressed as median with interquartile range (IQR) and analyzed using the Mann–Whitney U test. Categorical variables were presented as frequencies or percentages and compared using the chi-squared test.

A univariate binary regression analysis was performed to identify echocardiographic and radiological variables potentially associated with the need for PD across the entire patient cohort. Metric data underwent z-transformation to enhance comparability. Factors with a *p*-value of ≤0.100 in univariate analysis were included in a subsequent multivariable binary logistic regression to determine independent predictors of PD, with backward elimination used to refine the model.

To evaluate the predictive power of aortic valve maximum velocity (AV Vmax) in determining the need for PD, a receiver-operating characteristic (ROC) curve was generated, calculating the area under the curve (AUC), sensitivity, specificity, and Youden Index (YI).

A Kaplan–Meier survival analysis was conducted to explore differences in 30-day to 5-year mortality between patients who underwent PD and those who did not, with corresponding log-rank tests and risk numbers included.

Finally, an additional binary logistic regression analysis was performed to assess whether PD was associated with an increased risk of adverse clinical events.

A *p*-value of <0.050 was considered to indicate statistical significance in all analyses.

## 3. Results

The overall rate of post-dilatation in our study population was 11.5% (Table 1). The baseline characteristics (Table 1) highlight significant differences between patients who required PD after TAVI and those who did not. A smaller proportion of men underwent PD (40.3% vs. 50.6%, *p* = 0.113). Hemodynamic parameters were notably different, with the PD group exhibiting a higher AV MPG (47.0 ± 17.3 mmHg vs. 44.0 ± 11.8 mmHg, *p* < 0.001); aortic valve maximum pressure gradient (AV MAX) (82.0 ± 20.5 mmHg vs. 74.0 ± 19.0 mmHg, *p* < 0.001); and AV Vmax (4.6 ± 0.6 m/s vs. 4.3 ± 0.5 m/s, *p* < 0.001). Radiological factors, such as AVCS (4123.5 ± 2264.3 AU vs. 2813.5 ± 2060.0 AU), AVCi (2406.0 ± 1060.6 AU/m^2^ vs. 1562.7 ± 1114.4 AU/m^2^), and AVCd (865.7 ± 299.3 AU/cm^2^ vs. 598.2 ± 376.4 AU/cm^2^), demonstrated highly significant differences (*p* < 0.001 each) between the groups. Pre-dilatation of the native aortic valve immediately before implantation of the transcatheter valve prosthesis at operators’ discretion was performed in 11 patients (2.1%) within the group without PD, and in 1 patient (1.5%) within the PD group (0.732). Due to these small numbers, we refrained from further analyses in this regard.

The binary logistic regression analysis (Table 2) revealed that, in the univariate model, AV MPG, AV MAX, AV Vmax, AVCS, AVCi and AVCd were significantly associated with an increased likelihood of PD. However, in the multivariable analysis, only AV Vmax with an odds ratio (OR) of 1.424, a 95% confidence interval (CI): 1.039–1.950; *p* = 0.028 and an AVCd with OR: 1.618, 95% CI: 1.227–2.132; *p* = 0.001 remained an independent predictor for PD, while other parameters, including AV MAX, AV MPG, and AVCS, lost their significance.

**Figure 1:**
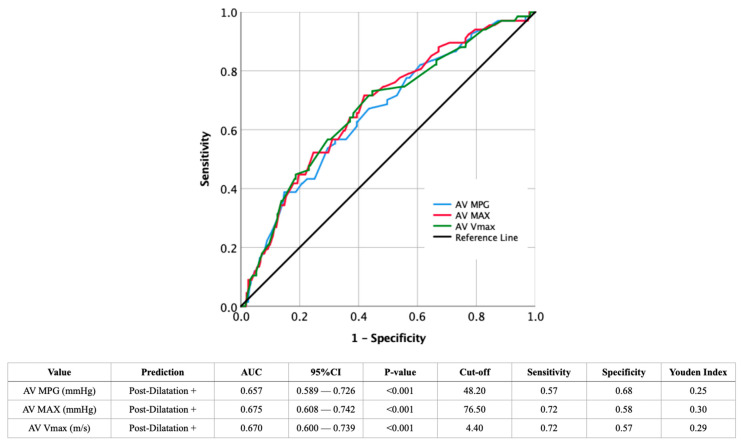
AUROC analysis of echocardiographic criteria for discrimination between the presence (+) or absence (−) of post-dilatation. CI: confidence interval; AUC: area under the curve; AV MAX: aortic valve maximum pressure gradient AV MPG: aortic valve mean systolic pressure gradient; AV Vmax: aortic valve maximal systolic transvalvular flow velocity.

The AUROC curves for AV MPG, AV MAX, and AV Vmax were analyzed to predict PD (Figure 1). The AV MAX demonstrated the highest AUC (0.675, 95% CI: 0.608–0.742, *p* < 0.001) with a sensitivity of 0.72 and a specificity of 0.58, at a cut-off of 76.50 mmHg. This was followed by AV Vmax, which had an AUC of 0.670 (95% CI: 0.600–0.739, *p* < 0.001), a sensitivity of 0.72, and a specificity of 0.57 at a cut-off of 4.40 m/s. The AV MPG showed the lowest AUC of 0.657 (95% CI: 0.589–0.726, *p* < 0.001), with a sensitivity of 0.57 and a specificity of 0.68 at a cut-off of 48.20 mmHg. The YI, which represents the optimal balance between sensitivity and specificity, was highest for AV MAX (0.30), followed by AV Vmax (0.29), and AV MPG (0.25).

**Figure 2:**
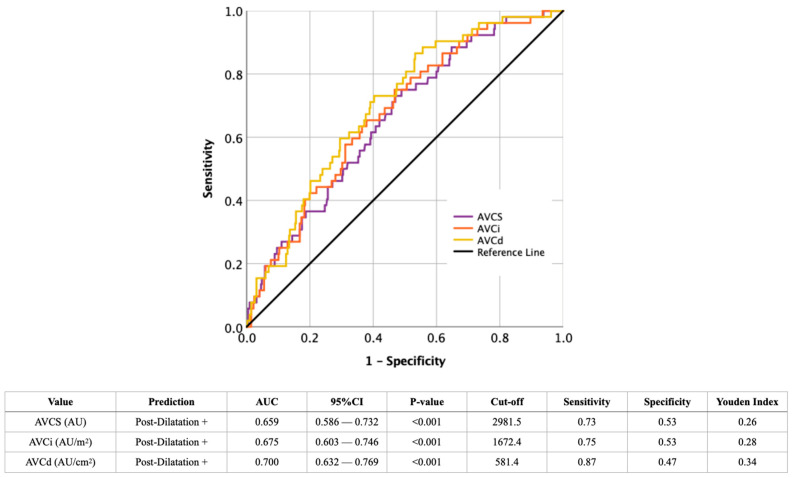
AUROC-analysis of radiological criteria for discrimination between the presence (+) or absence (−) of post-dilatation. CI: confidence interval; AU: Agatston unit; AUC: area under the curve; AVCd: aortic valve calcium density; AVCi: aortic valve calcium index; AVCS: aortic valve calcium score.

The AUROC analysis of AVCS, AVCi, and AVCd for predicting PD is shown in Figure 2. AVCd exhibited the best discriminative ability, with an AUC of 0.700 (95% CI: 0.632–0.769, *p* < 0.001), achieving a sensitivity of 87% and a specificity of 47% at a cut-off of 581.4 AU/cm^2^. AVCi had a slightly lower AUC of 0.675, while AVCS presented the lowest AUC (0.659). AVCd also had the highest YI (0.34), suggesting that it is the most effective radiological predictor.

**Figure 3:**
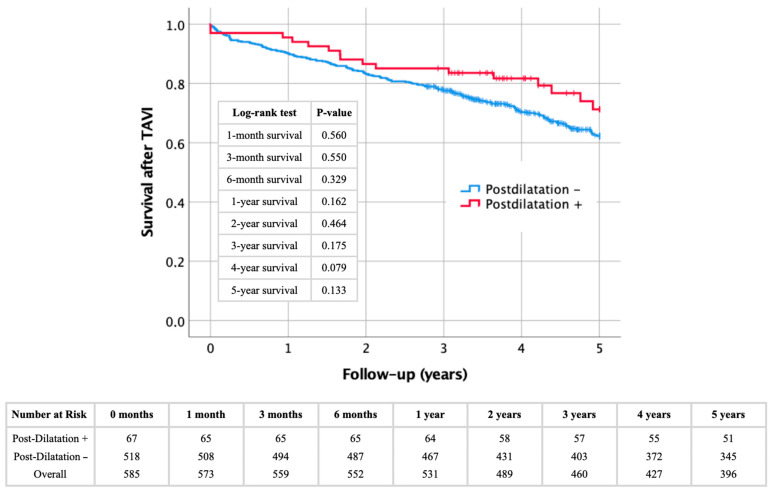
Kaplan–Meier curve with corresponding log-rank tests regarding overall short- and mid-term survival in patients with the presence (+) or absence (−) of post-dilatation after transcatheter aortic valve implantation.

A Kaplan–Meier analysis was performed to evaluate short- and mid-term survival in patients undergoing TAVI, comparing those who received PD to those who did not. As shown in the Kaplan–Meier survival curve (Figure 3), there is no statistically significant difference between the groups across the following time points: 1 month (*p* = 0.560); 3 months (*p* = 0.550); 6 months (*p* = 0.329); 1 year (*p* = 0.162); 2 years (*p* = 0.464); 3 years (*p* = 0.175); 4 years (*p* = 0.079); and 5 years (*p* = 0.133).

The occurrence of major adverse events following TAVI is presented in Table 3. No significant differences were found between the groups for specific adverse outcomes such as major vascular complications (0.0% vs. 3.5%, *p* = 0.121), pacemaker implantation (7.5% vs. 13.1%, *p* = 0.187), or stroke incidence (1.5% vs. 2.7%, *p* = 0.554).

The univariate binary logistic regression analysis (Table 4) revealed no significant associations between PD and individual outcomes, such as major vascular complications (odds ratio, OR: 0.000, 95% CI: 0.000, *p* = 0.998), pacemaker implantation (OR: 0.534, 95% CI: 0.207–1.375, *p* = 0.193), or stroke (OR: 0.544, 95% CI: 0.070–4.207, *p* = 0.560), indicating no significant elevation in these specific complications.

## 4. Discussion

### 4.1. Predicting the Probability of Post-Dilatation from Imaging Findings

Aortic valve calcium load: Aortic valve calcium has long been recognized as a potential predictor of TAVI procedural complications such as bioprosthesis underexpansion, paravalvular regurgitation, valve malpositioning, conduction disturbances, and annular rupture [12,13]. Since the underexpansion of bio-prosthesis, with or without paravalvular leakage, is the main indication for PD, the amount of aortic valvular calcium can be expected to predict the need for PD. While previous studies have suggested that a higher AVCS would increase the likelihood of PD, in our analysis, surprisingly, AVCS did not significantly predict the rate of PD. However, AVCd emerged as a stronger predictor, as shown by binary logistic regression and AUROC analysis. These results suggest that AVCd, which considers the concentration of calcium over the annulus area (“density”), may better reflect the mechanical challenges of valve deployment than the overall calcification score AVCS, which depends on local and systemic anatomical characteristics, including body size.

One possible explanation for the potentially diminished role of AVCS in predicting PD over the years could be improvements in self-expanding TAVI technology such as the then-current Evolut R and Evolut Pro devices used in this study. These devices apply consistent radial force even after deployment, which may allow for more complete valve expansion, even in heavily calcified settings. This could reduce the need for additional balloon expansion, even in the presence of significant calcification [3,14], which would make AVCS less predictive of PD.

Our findings contribute to a growing body of evidence suggesting that, while AVCS is useful in predicting outcomes, like paravalvular regurgitation, it may not be as predictive of the need for PD. The relevance of AVCd in this context underlines the need to reconsider the role of the absolute degree of calcification in procedural planning. Instead, future research could focus on identifying specific calcification characteristics, such as density or regional distribution within the aortic valve leaflets, which are likely to better predict PD risk, rather than relying solely on the overall calcification score.

Transvalvular flow velocity: Our study identified AV Vmax ≥ 4.0 m/s pre-TAVI as a significant independent predictor of the need for PD, with higher AV Vmax values correlating with an increased likelihood of requiring PD. This finding supports the notion that elevated AV Vmax may predict suboptimal valve deployment, necessitating PD to optimize valve function, minimize residual gradients [15], and reduce PVR [16]. The fact that AV Vmax, but not AVCS, is associated with PD suggests that other factors not directly related to the absolute calcium load—such as fibrotic components in the AS, or the distribution of valve calcification—play an important role in the development of underexposing and PVR, which represent the major indications for PD. Although AV Vmax shows (moderate) discriminative power in predicting the need for PD, as demonstrated by the AUROC analysis, its relatively low specificity suggests that it should not be used in isolation. Integrating AV Vmax with other echocardiographic parameters, such as aortic annulus dimensions, LVOT morphology, or LVOT calcium load, could enhance predictive accuracy. However, such an approach cannot be recommended at present and should be the subject of further studies.

### 4.2. Impact of Post-Dilatation on Procedural and Longterm Outcomes

Procedural outcomes: One of the most intriguing findings of our study was that PD was not associated with an elevation in adverse events. This counterintuitive result is contrary to the perception from earlier years in the TAVI era that PD could increase the risk of complications, including cerebrovascular events, conduction disturbances requiring pacemaker implantation, and annular rupture [6,17]. However, our findings align with studies that have reported similar outcomes [18,19]. For instance, a more recent study by Sanz Sánchez et al. [20] observed that PD in newer-generation self-expanding valves did not increase the risk of adverse events, suggesting that the more current generations of TAVI devices may offer improved safety profiles during PD.

Long-term outcomes: We found no significant survival difference between the group with PD and the group without PD over a period of five years. Despite concerns about premature valve degeneration due to the additional mechanical stress of the bioprosthesis during PD, there are few studies in the literature on long-term survival and other clinical endpoints after TAVI with PD. By far the longest follow-up period was again reported by Sanz Sanchez et al. [20], who tracked overall mortality over 6 years in a relatively large number of patients and also found no significant difference. The study also examined echocardiographic endpoints with regard to valve degeneration in the two groups—also without a significant difference.

### 4.3. Clinical Relevance

In line with previous studies, our study confirms the clinical experience of recent years that PD is not associated with a significantly increased risk in the context of contemporary TAVI procedures, provided that an appropriate clinical risk–benefit analysis is performed. The results thus support the concept of striving for an optimal implantation result—including PD, if necessary—which is particularly important in view of the increasing number of relatively young patients undergoing TAVI. In view of this, the pre-procedural assessment of the need for PD may no longer be as important as it was in previous years. However, particularly in high-risk patients, this will continue to be an aspect that will play an important role in the planning of TAVI—and in some cases possibly also in the decision for or against valve intervention. In this sense, our study can provide valuable information to those involved in heart team decisions.

### 4.4. Limitations

Despite providing valuable insights, our study has several limitations.

1. The retrospective design limits the ability to establish causal relationships between variables and the need for PD. Although significant associations were identified, prospective studies are required to confirm these findings and assess their real-world applicability. We focused on key echocardiographic and radiological factors, but other potential predictors, such as specific anatomical features or calcium distribution, were not analyzed;
2. The study was conducted at a single center with a relatively homogeneous patient population and small sample size, limiting the statistical power of our analyses and the generalizability to more diverse groups. Patient selection, procedural expertise, and device-specific factors might have influenced the outcomes. Larger, multicenter studies are needed to validate the findings and to establish whether they are transferable to other clinical and procedural settings;
3. The use of second- and third-generation self-expanding TAVI devices may not fully reflect the performance of newer systems, such as the Evolut Fx, currently in use; the findings are not generalizable to other self-expanding platforms with different designs and radial strengths such as the Acurate or Navitor systems. Of course, our results cannot be applied to balloon-expanded TAVI systems, which generally have significant lower post-dilatation rates compared with self-expanding systems due to their different deployment technology;
4. As a systematic limitation, it should be pointed out that the multitude of anatomical, morphological, functional and clinical criteria that contributed to the operators’ decisions whether or not to post-dilate—which were inherently made intraprocedurally and under limited time resources—could not be assessed in this retrospective analysis.

## 5. Conclusions

In our study, AVCS as a measure of absolute valvular calcium load did not predict the likelihood of PD during TAVI procedure with a self-expanding bioprosthesis, whereas aortic valve calcium density and AV-Vmax were identified as significant predictors of PD. PD was not associated with an increase in adverse events. Our study therefore suggests that PD can be performed safely when indicated. Longterm mortality was also not affected by PD.

## Figures and Tables

**Table 1:** Baseline characteristics of study cohort.

	Post-Dilatation+	Post-Dilatation−	*p*
*n*/%	67/11.5	518/88.5	-
Sex (male)—*n*/%	27/40.3	262/50.6	0.113
Age (years)—mean ± SD	80.4 ± 5.4	82.3 ± 5.0	0.004
Height (cm)—mean ± SD	166.4 ± 8.2	168.1 ± 9.8	0.172
Weight (kg)—mean ± SD	71.8 ± 14.2	73.1 ± 14.5	0.497
BMI (kg/m^2^)—mean ± SD	25.9 ± 4.5	25.8 ± 4.4	0.825
BSA (m^2^)—mean ± SD	1.8 ± 0.2	1.8 ± 0.2	0.463
LVEF (%)—median ± IQR	55.0 ± 15.0	55.0 ± 10.0	0.417
IVSD (mm)—median ± IQR	14.0 ± 3.3	13.0 ± 2.4	0.265
LVEDD (mm)—median ± IQR	4.6 ± 1.0	4.6 ± 0.9	0.660
AV MPG (mmHg)—median ± IQR	47.0 ± 17.3	44.0 ± 11.8	<0.001
AV MAX (mmHg)—median ± IQR	82.0 ± 20.5	74.0 ± 19.0	<0.001
AV Vmax (m/s)—median ± IQR	4.6 ± 0.6	4.3 ± 0.5	<0.001
Annulus Diameter (mm)—median ± IQR	25.0 ± 2.3	25.0 ± 3.0	0.850
Annulus Area (cm^2^)—median ± IQR	467.5 ± 87.0	472.0 ± 124.3	0.797
AVCS (AU)—median ± IQR	4123.5 ± 2264.3	2813.5 ± 2060.0	<0.001
AVCi (AU/m^2^)—median ± IQR	2406.0 ± 1060.6	1562.7 ± 1114.4	<0.001
AVCd (AU/cm^2^)—median ± IQR	865.7 ± 299.3	598.2 ± 376.4	<0.001
Pre-dilatation performed—*n*/%	1/1.5	11/2.1	0.732

AVCd: aortic valve calcium density; AVCi: aortic valve calcium index; AVCS: aortic valve calcium score; AV MAX: aortic valve maximum pressure gradient; AV MPG: aortic valve mean systolic pressure gradient; AV Vmax: aortic valve maximal systolic transvalvular flow velocity; BMI: body mass index; BSA: body surface area; IQR: interquartile range; IVSD: interventricular septal thickness; LVEDD: left ventricular end diastolic diameter; LVEF: left ventricular ejection fraction; SD: standard deviation.

**Table 2:** Univariate and multivariable binary logistic regression regarding echocardiographic and radiological parameters in the presence of post-dilatation after TAVI.

Post-Dilatation Binary Logistic Regression	Univariate		Multivariable	
	Odds Ratio (95% CI)	*p*	Odds Ratio (95% CI)	*p*
LVEF	0.789 (0.419–1.487)	0.464		
IVSD	1.036 (0.824–1.304)	0.762		
LVEDD	0.613 (0.147–2.561)	0.502		
AV MPG	1.607 (1.261–2.049)	<0.001	0.788 (0.470–1.321)	0.366
AV MAX	1.679 (1.314–2.145)	<0.001	1.187 (0.455–3.100)	0.726
AV Vmax	1.780 (1.361–2.328)	<0.001	1.424 (1.039–1.950)	0.028
Annulus Diameter	0.961 (0.725–1.274)	0.783		
Annulus Area	0.984 (0.743–1.304)	0.911		
AVCS	1.636 (1.269–2.111)	<0.001	0.953 (0.531–1.710)	0.872
AVCi	1.680 (1.293–2.184)	<0.001	1.248 (0.370–4.215)	0.721
AVCd	1.794 (1.387–2.321)	<0.001	1.618 (1.227–2.132)	0.001

AVCd: aortic valve calcium density; AVCi: aortic valve calcium index; AVCS: aortic valve calcium score; AV MAX: aortic valve maximum pressure gradient; AV MPG: aortic valve mean systolic pressure gradient; AV Vmax: aortic valve maximal systolic transvalvular flow velocity; CI: confidence interval; IVSD: interventricular septal thickness; LVEDD: left ventricular end diastolic diameter; LVEF: left ventricular ejection fraction.

**Table 3:** Specific outcome of study cohort regarding presence or absence of post-dilatation.

	Post-Dilatation+	Post-Dilatation−	*p*
Major Vascular Complications after TAVI	0/0.0	18/3.5	0.121
Pacemaker after TAVI—*n*/%	5/7.5	68/13.5	0.187
Stroke after TAVI—*n*/%	1/1.5	14/2.7	0.554

TAVI: transcatheter aortic valve implantation.

**Table 4:** Univariate binary logistic regression regarding outcome parameters in the presence of post-dilatation after TAVI.

Post-Dilatation Binary Logistic Regression	Univariate	
	Odds Ratio (95% CI)	*p*
Major Vascular Complications after TAVI	0.000 (0.000–.)	0.998
Pacemaker after TAVI	0.534 (0.207–1.375)	0.193
Stroke after TAVI	0.544 (0.070–4.207)	0.560

CI: confidence interval; TAVI: transcatheter aortic valve implantation.

## Data Availability

The datasets analyzed during the current study were retrieved from the ORBIS electronic medical records system and a separate medical archive system at the University Hospital Salzburg. Due to patient privacy and institutional regulations, the datasets are not publicly available but are available from the corresponding author upon reasonable request and with appropriate ethical approval.

## Abbreviations

The following abbreviations are used in this manuscript:

AS	Aortic Stenosis
AV	Aortic Valve
AV MAX	Aortic Valve Maximum Pressure Gradient
AV MPG	Aortic Valve Mean Systolic Pressure Gradient
AV Vmax	Aortic Valve Maximal Systolic Transvalvular Flow Velocity
AVCS	Aortic Valve Calcium Score
AVCi	Aortic Valve Calcium Index
AVCd	Aortic Valve Calcium Density
AUC	Area Under the Curve
AUROC	Area Under the Receiver Operating Characteristic Curve
BSA	Body Surface Area
BMI	Body Mass Index
CI	Confidence Interval
CT	Computed Tomography
CTA	Computed Tomography Angiography
ECG	Electrocardiogram
ESC	European Society of Cardiology
IQR	Interquartile Range
IVSD	Interventricular Septal Thickness
LVEF	Left Ventricular Ejection Fraction
LVEDD	Left Ventricular End-Diastolic Diameter
MDCT	Multi-Detector Computed Tomography
OR	Odds Ratio
PD	Post-Dilatation
PVR	Paravalvular Regurgitation
ROC	Receiver Operating Characteristic
SD	Standard Deviation
TEE	Transesophageal Echocardiography
TTE	Transthoracic Echocardiography
TAVI	Transcatheter Aortic Valve Implantation
VARC-3	Valve Academic Research Consortium-3
YI	Youden Index
